# Comparison of neurological health outcomes between two adolescent cohorts exposed to pesticides in Egypt

**DOI:** 10.1371/journal.pone.0172696

**Published:** 2017-02-23

**Authors:** Ahmed A. Ismail, Matthew R. Bonner, Olfat Hendy, Gaafar Abdel Rasoul, Kai Wang, James R. Olson, Diane S. Rohlman

**Affiliations:** 1 Department of Occupational and Environmental Health, College of Public Health, University of Iowa, Iowa City, Iowa, United States of America; 2 Community Medicine and Public Health Department, Faculty of Medicine, Menoufia University, Shebin El-Kom, Egypt; 3 Department of Epidemiology and Environmental Health, State University of New York, Buffalo, New York, United States of America; 4 Department of Clinical Pathology, National Liver Institute, Menoufia University, Shebin El-Kom, Egypt; 5 Department of Biostatistics, College of Public Health, University of Iowa, Iowa City, Iowa, United States of America; 6 Department of Pharmacology and Toxicology, State University of New York, Buffalo, New York, United States of America; 7 Oregon Institute for Occupational Health Sciences, Oregon Health & Science University, Portland, Oregon, United States of America; Universiteit Gent, BELGIUM

## Abstract

Pesticide-exposed adolescents may have a higher risk of neurotoxic effects because of their developing brains and bodies. However, only a limited number of studies have addressed this risk among adolescents. The aim of this study was to compare neurological outcomes from two cohorts of Egyptian adolescents working as pesticide applicators. In 2005 and 2009, two cohorts of male adolescents working as pesticide applicators for the cotton crop were recruited from Menoufia Governorate, Egypt. The same application schedule and pesticides were used at both times, including both organophosphorus, and pyrethroid compounds. Participants in both cohorts completed three neurobehavioral tests, health and exposure questionnaires, and medical and neurological screening examinations. In addition, blood samples were collected to measure butyryl cholinesterase (BChE) activity. Pesticide applicators in both cohorts reported more neurological symptoms and signs than non-applicators, particularly among participants in the 2005 cohort (OR ranged from 1.18 to 15.3). Except for one test (Trail Making B), there were no significant differences between either applicators or non-applicators of both cohorts on the neurobehavioral outcome measures (p > 0.05). The 2005 cohort showed greater inhibition of serum BChE activity than the 2009 cohort (p < 0.05). In addition, participants with depressed BChE activity showed more symptoms and signs than others without BChE depression (p < 0.05). Our study is the first to examine the consistency of health outcomes associated with pesticide exposure across two cohorts tested at different times from the same geographical region in rural Egypt. This similar pattern of findings across the two cohorts provides strong evidence of the health impact of exposure of adolescents to pesticides.

## 1. Introduction

Adolescents are occupationally exposed to pesticides while performing a range of tasks during the pesticide application process. These tasks range from applying with a backpack sprayer, mixing and loading pesticides, cleaning the equipment or holding signs to mark the edges of the field during the application process [1]. Pesticide application to the cotton crop in Egypt is managed and regulated by the Egyptian Ministry of Agriculture. Pesticides are applied during the summer at 4 time-points according to the life cycles of different cotton worms: cotton bollworms (pink and spiny) and cotton leafworms [2]. Early in the growing season, a biological bacterial insecticide is applied as a growth promoter, followed by 3 cycles of pesticide application throughout July and August to control cotton worm infestations. The applied insecticides include organophosphorus (primarily chlorpyrifos) and pyrethroid compounds [1].

Previous research examining long-term occupational exposure among adults, without previous poisoning, has demonstrated increased reporting of a wide range of neurological symptoms and signs, most commonly fatigue, headache, blurred vision, dizziness [3–5], but also depression, difficulty with concentration or memory, irritability, and numbness [6, 7]. Others have reported nerve function abnormalities, paresthesia, increased vibration sensitivity, balance difficulties, tremors, staggering and weakness, hyper-reflexia and loss of muscle strength in legs or arms, and difficulty in moving fingers or grasping objects [4, 8, 9]. Neurobehavioral performance was also examined among adult pesticide workers; it was found that workers exposed to organophosphorus (OP) pesticides demonstrate deficits in response speed and coordination, sustained attention, visual perception, and memory [10, 11]. More years working in agriculture and handling pesticides is associated with increased neurobehavioral deficits [12, 13]. Few studies have addressed these outcomes among adolescent pesticide applicators [1, 14–17]. The most commonly reported neurobehavioral deficits among adolescents were motor speed and coordination, information processing speed and executive functioning, attention, and memory [1, 14, 16]. Adolescents working in agriculture showed more neurological symptoms, e.g. headache, tremors, insomnia, dizziness, irritability, and numbness, than adolescents not working in agriculture [15, 17].

Cholinesterase activity as a biomarker of effect is often used to characterize exposure to OP pesticides, where the common mechanism of OP neurotoxicity is the inhibition β-cholinesterases: acetyl cholinesterase (AChE) and butyryl cholinesterase (BChE), with a more sensitivity of BChE [18]. Several studies with adults have found that cholinesterase activity decreased after exposure [19–21]. Other studies reported lower cholinesterase levels among exposed participants than control participants [11, 22]. Studies with adolescents have also demonstrated lower cholinesterase activity among adolescent applicators compared to non-applicators [1, 16, 18]. Although numerous studies have examined the relationship between neurological symptoms reporting and cholinesterase activity [1, 4–7, 17, 21, 23], there is a scarcity of the studies that interested in studying this relationship among adolescents.

Adolescents may be at greater risk from pesticide exposure than adults because of their still developing bodies [24, 25]; Furthermore, they may perform agricultural tasks that put them at risk for exposure, particularly in countries where there are few restrictions addressing children’s work in agriculture [1, 19]. This demonstrates a need to examine the impact of exposure to pesticides on neurological outcomes among adolescents occupationally working in agriculture. In order to advance our understanding, it is important to look for converging evidence, replicating the neurological and neurobehavioral findings of similar cohorts who are exposed in the same way and evaluating them using the same tools is needed to confirm the relationship between exposure of adolescents to pesticides and the deleterious effects of pesticides.

Adverse effects associated with pesticide exposure were first identified in a cohort of adolescents in 2005 [1]. A second study in 2009 was conducted to determine if similar effects were found and to evaluate additional outcome measures [16]. Similar application schedules and pesticides were applied at both time points. The goal of the current work was to examine the impact of pesticide exposure on health outcomes, including: neurobehavioral symptoms and signs, neurobehavioral performance, and butyryl cholinesterase (BChE) activity among the two cohorts of participants examined in 2005 and 2009, and evaluate whether these effects are consistent or can be replicated over time.

## 2. Methods

### 2.1. Participants

Participants for both cohorts were recruited from Menoufia Governorate, and testing was carried out in August of 2005 and 2009, at the end of the pesticide application season. The season begins in June and ends in August. During this time, approximately 10 adolescents from each village were hired by the local stations of the Ministry of Agricultural to apply pesticides to the cotton crop under supervision of adult agriculture engineers and employees. Adolescents hired by the Ministry of Agriculture, between 12 and 18 years of age, were recruited in 2005 and 2009 (N = 41 in 2005 and N = 21 in 2009). There was a high response rate at both time periods (89.3% in 2005 and 91.3% in 2009). Adolescents who had never worked in the cotton fields was also recruited through friends and relatives of the applicator adolescents (N = 38 in 2005 and N = 20 in 2009) as a comparison group. Non-applicators lived in the same community as applicators and attended the same schools, but were not interested in working as pesticide applicators. The pesticide application process was the same at both years and is described in detail elsewhere [1]. The protocol and consent forms used in this study were approved by the Oregon Health & Science University (USA) and Menoufia University (Egypt) Institutional Review Boards. Participants and their legal guardians gave written informed consent prior to enrollment.

### 2.2. Procedure

Both cohorts, 2005 and 2009, completed questionnaires, provided a blood sample and performed a battery of neurobehavioral tests. The 2009 participants also provided a urine sample for the measurement of 3,5,6-trichloro-2-pyridinol (TCPy); a specific metabolite for chlorpyrifos, the primary pesticide applied. Although a subset of tests was the same at both years, in 2005, all neurobehavioral tests were traditional tests from the Wechsler Adult Intelligence Scale—Revised (WAIS-R). In 2009, a series of computerized tests from the Behavioral Assessment and Research System (BARS) replaced some of the individually administered tests (Table 1). Data collection methods are described in brief below.

**Table 1:** Neurobehavioral functions assessed and the tests administered for both cohorts (2005 and 2009).

Functions	Tests	2005	2009	Format
Memory	Match To Sample; MTS ^a^		√	Computer
	Serial Digit Learning; SDL ^a^		√	Computer
	Benton Visual Retention; BVRT ^b^	√	√	Paper-pencil
Attention/Short memory	WAIS-R Digit Span; DS ^b^	√		Paper-pencil
	Digit Span Test; DST ^a^		√	Computer
	WAIS-R Arithmetic ^b^	√		Paper-pencil
Sustained Attention	Continuous Performance; CPT ^a^		√	Computer
Motor Speed/Coordination	Finger Tapping; TAP ^a^		√	Computer
Information Processing Speed	Simple Reaction; SRT ^a^		√	Computer
Visual Motor	WAIS-R Digit Symbol ^b^		√	Paper-pencil
	Symbol Digit; SDT ^a^		√	Computer
	Trail Making A & B ^b^	√	√	Paper-pencil
Perception	WAIS-R Block Design ^b^	√	√	Manual

#### 2.2.1. Questionnaires

For both cohorts, adolescents with the assistance of their parents, completed a questionnaire describing their medical and work history, including information about their exposure to pesticides. The questionnaire included items asking about the frequency of neurological symptoms e.g. headache, pain, and fatigue. In 2009, detailed questions were added to the questionnaire to get a complete profile of exposure to pesticides at work, home, and in family fields.

#### 2.2.2. Medical examination

Detailed clinical medical examinations and complete neurological examinations were administered by specialists during both years. These examinations included assessment of the following signs among study cohorts: tremors, and neurological incoordination, in addition to any abnormalities in ankle and knee reflexes, superficial and deep sensations, or in muscle power.

#### 2.2.3. Neurobehavioral test battery

Age appropriate versions of tests from the Wechsler Adult Intelligence Scale—Revised (WAIS-R; the current version available at the time) [26], validated in an Arabic-speaking population [27], were used to assess neurobehavioral function. In addition, the 2009 cohort also completed computerized neurobehavioral tests from the Behavioral Assessment and Research System (BARS; Table 1) [28]. All test instructions were translated into Arabic. Examiners read the instructions to participants for non-computerized tests, while for the computerized tests, instructions were presented in Arabic on the screen and also simultaneously delivered orally through headphones. Reliability and validity of BARS tests were approved for the Arabic speaking populations [16, 29]. For the purpose of the current study, the comparison between both cohorts was done for the three neurobehavioral tests that were administered identically to both cohorts.

#### 2.2.4. Butyryl cholinesterase activity

Cholinesterase enzyme activity, as a biomarker of effect chlorpyrifos, was evaluated at the end of the application season for both study cohorts. The Weber method [30] was used to measure the serum cholinesterase enzyme (Butyryl cholinesterase; BChE) in 2005 through a standard laboratory kit (Test-combination Boehringer Mannheim GmbH Diagnostica). In 2009, Butyryl cholinesterase activity was measured in whole blood using the EQM Test-Mate kit (EQM Research, Cincinnati, OH, USA) [31], which is based on the Ellman method [32].

#### 2.2.5. Data analysis

SPSS version 23 was used for data analysis. Analysis of Variance (ANOVA) test was employed to test the difference between cohorts (2005 and 2009), across job categories (applicators and non-applicators), and the interaction between cohorts and job categories. Odds ratios (ORs) and their confidence intervals were used to estimate the risk of developing symptoms and signs between groups. These ORs for both cohorts were contrasted to test their homogeneity using chi-square tests and p-values. When the results were homogenous, Mantel Haenszel common OR was used to test the significance of ORs over the two study cohorts. Regression analysis was performed to examine the differences in performance on the three neurobehavioral tests administered to both cohorts, between applicators and non-applicators across the two cohorts. Differences of adjusted means and their standard errors are presented for the neurobehavioral tests controlling for age and years of education. The differences in number of symptoms between cohorts, job categories, and also beween depressed and non-depressed BChE activity participants were analyzed using the Mann-Whitney test. Chi-square test was used to examine the differences between cohorts (within job categories) and between job categories (within cohorts) among the participants with depressed BChE activity. Pearson correlation was run to test the correlation between neurobehavioral tests and BChE activity for both cohorts.

## 3. Results

### 3.1. Characteristics of participants

Table 2 describes the demographic characteristics of participants in both the 2005 and 2009 cohorts. Tests compare the mean responses between cohorts (within job categories), between job categories (within cohorts), and modification of differences between job categories over time (interaction effect). Means and standard deviations (SD) were reported and compared with ANOVA test. Age was not significantly different between all groups, averaging 15.4 years overall. The 2009 participants had more years of education than the 2005 participants, but there were no differences in years of education between applicators and non-applicators of both cohorts. While the 2005 applicators worked significantly more years in agriculture than the 2009 applicators (p < 0.001), the difference in days worked in pesticide application in 2005 and 2009 was not significantly different between applicators of both cohorts (p = 0.08).

**Table 2:** Comparison of demographic and exposure characteristics of applicators and non-applicators in 2005 (n = 79), and 2009 (n = 41).

Characteristics	2005		2009		P-value		
	Non-applicators (n = 38) Mean (SD)	Applicators (n = 41) Mean (SD)	Non-applicators (n = 20) Mean (SD)	Applicators (n = 21) Mean (SD)	Cohort^a^	Job category^b^	Interaction^c^
**Age (y)**	15.4 (1.7)	15.2 (1.7)	15.5 (1.5)	15.5 (2.1)	0.6	0.8	0.8
**Education (y)**	7.8 (3.8)	6.8 (4.2)	9.5 (1.5)	9.3 (1.5)	< 0.001	0.2	0.4
**Days worked**		22.4 (6.9)		18.9 (7.4)	0.08		
**Years worked**		5.5 (2.3)		2.1 (1.0)	< 0.001		

^a,^ comparison between 2005 and 2009 cohorts

^b,^ comparison between applicators and non-applicators across the two years of the study

^c,^ Interaction between job category and cohort

### 3.2. Neurological manifestations

Table 3 shows that applicators in both cohorts reported higher frequencies of neurological symptoms and signs than non-applicators, but only the 2005 applicators reported significantly more neurological symptoms and signs than non-applicators (20 out of the 25 manifestations had significant 95% CI). A test of homogeneity of ORs of both cohorts for each symptom was conducted to see if the ORs of both cohorts were different. Outcomes of the homogeneity OR test indicated that ORs of both cohorts for all symptoms are homogenous, p > 0.1 (Table 3, the 2^nd^ right column). In the last column of Table 3, the Mantel Haenszel common OR (M-H OR) is used for all symptoms and signs to examine the significance of common OR across the two cohorts. Common ORs are significant for most of the neurological symptoms and signs; this indicates higher frequencies of symptoms and signs among applicators than non-applicators. All reported ORs were unadjusted due the small sample sizes of both cohorts. The median number of symptoms and signs was also significantly higher among applicators than non-applicators (within cohorts, p = 0.03) and higher for the 2005 cohort than that of the 2009 cohort (p = 0.005) (Data not shown).

**Table 3:** Comparison of the neurological symptoms and signs between job categories in each cohort (OR and CI), and homogeneity evaluation of the comparisons across the two cohorts (X^2^, p-value) and the significance of the Mantel-Haenszel common odds ratio (M-H OR (95% CI).

Signs/Symptoms	2005			2009			X^2^ (1df), p	M-H OR (95% CI)
	Non-applicators (n = 38) N (%)	Applicators (n = 41) N (%)	OR (95% CI)	Non-applicators (n = 20) N (%)	Applicators (n = 21) N (%)	OR (95% CI)		
**Headache**	9 (23.7)	15 (36.6)	1.86 (0.70, 4.96)	2 (10.0)	4 (19.0)	2.12 (0.34, 13.1)	0.02, 0.90	1.92 (0.81, 4.54)
**Arthralgia**	9 (23.7)	11 (26.8)	1.18 (0.43, 3.27)	2 (10.0)	3 (14.3)	1.50 (0.22, 10.1)	0.047, 0.83	1.25 (0.51, 3.06)
**Pain**	6 (15.8)	8 (19.5)	1.29 (0.40, 4.14)	2 (10.0)	2 (9.5)	0.95 (0.12, 7.46)	0.07, 0.79	1.20 (0.44, 3.30)
**Fatigue**	3 (7.9)	15 (36.6)	6.73 (1.76, 25.7)	1 (5.0)	4 (19.0)	4.47 (0.45, 44.0)	0.09, 0.76	6.06 (1.91, 19.2)*
**Blurred vision**	2 (5.3)	14 (34.1)	9.33 (1.95, 44.6)	1 (5.0)	3 (14.3)	3.17 (0.30, 33.3)	0.59, 0.44	6.92 (1.92, 25.0)*
**Feeling depressed**	2 (5.3)	13 (31.7)	8.36 (1.74, 44.1)	1 (5.0)	4 (19.0)	4.47 (0.45, 44.0)	0.20, 0.66)	6.92 (1.91, 25.1)*
**Difficulty in concentration**	2 (5.3)	12 (29.3)	7.45 (1.54, 36.0)	0	1 (4.8)		0.13, 0.72	8.11 (1.69, 39.0)*
**Dizziness**	2 (5.3)	12 (29.3)	7.45 (1.54, 36.0)	0	0			
**Difficulty in understanding** ^a^	2 (5.3)	11 (26.8)	6.6 (1.36, 32.1)	1 (5.0)	2 (9.5)	2.00 (0.17, 24.0)	0.67, 0.41	4.86 (1.31, 18.0)*
**Troubles in remembering** ^b^	1 (2.6)	12 (29.3)	15.3 (1.88, 125)	1 (5.0)	4 (19.0)	4.47 (0.45, 44.0)	0.65, 0.42	9.56 (2.11, 43.4)*
**Feeling irritable**	1 (2.6)	11 (26.8)	13.6 (1.66, 111)	1 (5.0)	4 (19.0)	4.47 (0.45, 44.0)	0.52, 0.47	8.82 (1.93, 40.3)*
**Numbness**	1 (2.6)	9 (22.0)	10.4 (1.25, 86.6)	0	3 (14.3)		0.31, 0.58	14.0 (1.73, 114)*
**Superficial sensation abnormality**	2 (5.3)	12 (29.3)	7.45 (1.54, 36.0)	0	3 (14.3)		0.43, 0.51	9.44 (2.00, 44.6)*
**Knee reflex abnormality**	2 (5.3)	10 (24.4)	5.81 (1.18, 28.5)	0	1 (4.8)		0.17, 0.68	6.43 (1.32, 31.3)*
**Tremors**	2 (5.3)	8 (19.5)	4.36 (0.86, 22.0)	0	1 (4.8)		0.22, 0.64	4.95 (1.00, 24.5)*
**Incoordination**	1 (2.6)	10 (24.4)	11.9 (1.45, 98.5)	2 (10.0)	4 (19.0)	2.12 (0.43, 13.1)	1.63, 0.20	5.27 (1.43, 19.4)*
**Ankle reflex abnormality**	1 (2.6)	6 (14.6)	6.34 (0.73, 55.4)	0	1 (4.8)		0.15, 0.70	7.44 (0.87, 63.6)
**Muscle power abnormality**	1 (2.6)	5 (12.2)	5.14 (0.57, 46.2)	0	1 (4.8)		0.19, 0.66	6.21 (0.71, 54.0)
**Deep sensation abnormality**	0	1 (2.4)		0	0			

^a,^ Difficulty in understanding meanings of newspaper and books

^b,^ Troubles in remembering things observed by relatives

### 3.3. Neurobehavioral performance

There were no significant differences between applicators of both study cohorts and also no significant differences for non-applicators on the neurobehavioral tests after controlling for age and years of education, except that non-applicators in 2009 took a significantly longer time to complete the Trail Making-B test than non-applicators in 2005 (Table 4).

**Table 4:** Differences in neurobehavioral performance between applicators and non-applicators of both cohorts.

Neurobehavioral Test	Non-applicators Difference (95% CI)	Applicators Difference (95% CI)
**Block Design**	3.9 (-1.5, 9.3)	4.1 (-0.02, 8.2)
**Trail Making—A** ^a^	- 9.9 (-20.0, 0.3)	- 8.9 (-20.1, 2.2)
**Trail Making—B** ^a^	- 21.8 (-42.0, -1.7)*	- 8.7 (-24.5, 7.2)

^a,^ Differences represents (2005–2009) time in seconds. These are time measures tests, so, higher is worse

* p < 0.05

### 3.4. Butyryl cholinesterase activity of both cohorts of the study

**Fig 1:**
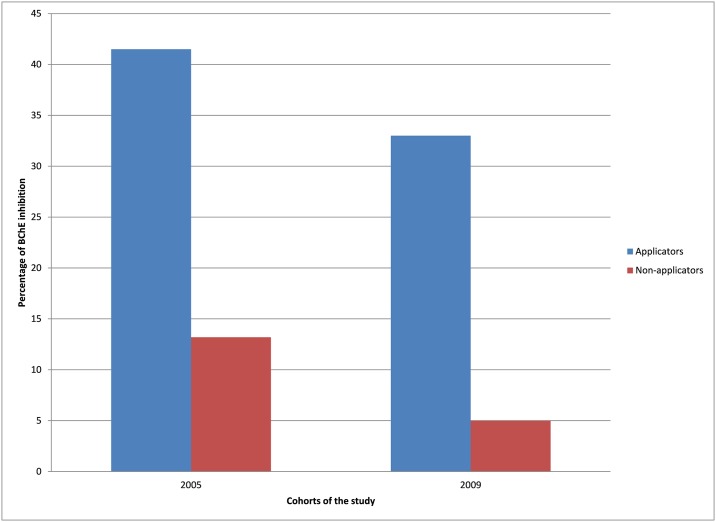
Percent of participants with depressed BChE activity relative to the normal ranges in 2005 and 2009.

The number of participants with BChE inhibition were compared across the study cohorts; however, different methods were employed to evaluate BChE activity in both cohorts. The range of normal BChE activity reported by kit manufacturers (3.33–7.03 IU/ml for the 2005 cohort [30], and 1.35–3.23 U/ml for the 2009 cohort [31]) were used to identify participants with depressed or low BChE activity relative to the respective normal range. For both cohorts, a greater percentage of applicators had depressed levels of cholinesterase, relative to the normal range, than non-applicators (Fig 1; 2005: chi-square = 7.9, p = 0.005; 2009: chi-square = 5.2, p = 0.02).

### 3.5. Relationship between neurological symptoms and signs and butyryl cholinesterase activity

Among participants in the 2005 cohort, participants with depressed BChE activity reported a greater number of neurological symptoms and signs relative to participants that were within the normal range of BChE activity (p = 0.006). This relationship was also observed when participants from the 2005 and 2009 cohorts were combined (p = 0.013). However, this difference between participants with depressed and normal BChE was not found for the 2009 cohort (p = 0.75) (Table 5).

**Table 5:** Median and interquantile range (IQR) of neurological symptoms and signs of the two cohorts according to BChE activity (in each cohort, depression in BChE activity was determined relative to normal ranges from the methods used).

	2005 (n = 79)		2009 (n = 41)		Total (n = 120)	
	Depressed BChE (n = 22)	Normal BChE (n = 57)	Depressed BChE (n = 8)	Normal BChE (n = 33)	Depressed BChE (n = 30)	Normal BChE (n = 90)
**Median**	4.0	0.0	0.0	0.0	1.5	0.0
**IQR**	10.2	2.0	2.2	1.0	9.2	1.0
**p-value** ^a^	0.006		0.75		0.013	

^a,^ P-value of Mann-Whitney test

### 3.6. Correlation between neurobehavioral performance and butyryl cholinesterase

**Fig 2:**
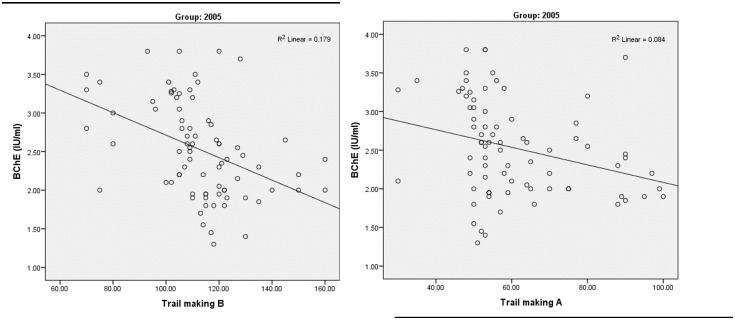
Correlation between Trail making test (A & B) and butyryl cholinesterase (BChE) levels in participants from the 2005 cohort.

Pearson correlation was used to examine the correlation between neurobehavioral tests and BChE activity for both study cohorts. A non-significant correlation between neurobehavioral outcomes in both study cohorts and BChE levels was found, except for Trail making A and B in the 2005 cohort. However, a significant negative correlation was present between the BChE level and Trail Making A (r = -0.29, p = 0.01), and also with Trail Making B (r = -0.42, p < 0.001) (Fig 2). This indicates impaired performance in these tests with more depression of BChE activity.

## 4. Discussion

Our study is the first to examine the consistency of health outcomes associated with pesticide exposure across two cohorts tested at different times from the same geographical region in rural Egypt. Pesticide applicators in both cohorts reported more neurological manifestations, neurobehavioral deficits, and BChE inhibition than non-applicators. The consistency of the findings over the two cohorts of the study provides further evidence of the neurological health effects of prolonged exposure to organophosphorus pesticides. The two cohorts were examined in 2005 and 2009 using a similar methodology with similar questionnaires, neurobehavioral testing, medical examination, and evaluation of BChE activity. During the two years of the study, applicators also applied the same types of pesticides, and followed the same schedule and methods of pesticide application. However, the 2005 cohort showed more neurological symptoms and signs, and neurobehavioral deficits, which in part may be due to more years of working in pesticide application and/or more extensive exposures, and fewer years of education.

Pesticide applicators from both cohorts reported more neurological symptoms and showed more signs than non-applicators of both study cohorts. Nevertheless, the significant ORs were obtained only among the 2005 cohort; the consistent higher frequencies of symptoms and signs among applicators were confirmed through the homogeneity of OR as shown by the chi-square test, and the significant common OR by the Mantel-Haenszel test (Table 3). The results of neurological signs and symptoms among adolescent pesticide applicators of both 2005 and 2009 cohorts agree with the findings of Egyptian studies that examined health hazards among adult pesticide applicators [3, 11], and applicators and farmworkers worldwide: licensed private applicators [6], Florida farmworkers [10], and farm residents [7] in the USA; irrigation workers in Ghana [33], Indonesian farmers [34], Sri Lankan farmers [4], Spanish greenhouse sprayers [35], Indian pesticide manufacturing workers [22], and Emirates farmworkers [36].

This consistency of neurobehavioral findings is also found through comparison of neurobehavioral tests applied identically to both cohorts: Block Design, Trail Making A and B, where applicators and non-applicators of both cohorts did not show any statistically significant difference except on the Trail Making B test (Table 4). Detailed comparisons of neurobehavioral performance of applicators and non-applicators for each cohort are presented in other publications [1, 16]. The outcomes of the current study confirm the findings of other studies which showed that functional domains most consistently affected by OP exposure include psychomotor and cognitive behavior [37]. These findings also were confirmed by a meta-analysis examining neurobehavioral performance among farmworkers and pesticide applicators [38].

Although pesticide applicators in 2009 did not show significantly more neurological symptoms and signs than the non-applicators, most likely due to the small sample size, this did not affect the homogeneity of OR measured by the chi-square test or the significant common OR by the Mantel-Haenszel test for the majority of the neurological symptoms and signs (Table 3). These findings indicate an increase in frequency of both neurological symptoms and signs among applicators compared to non-applicators, regardless of the examined cohort. The same results were obtained when comparing the median of the number of symptoms and signs experienced by each participant between applicators and non-applicators in each cohort and across the two cohorts (last row-Table 3). This association between neurobehavioral deficits and occupational exposure to OP pesticides among applicators of both cohorts is confirmed by the findings of non-significant differences between 2005 and 2009 cohorts in age and days worked in applying pesticides (Table 2), and also after controlling for both age and years of education in the regression model for neurobehavioral outcomes (Table 4). The relationship between OP exposure and neurobehavioral deficits is also strengthened because applicators from both cohorts used the same chemical compounds, the same method of pesticide application and almost the same work duration in both seasons.

In evaluating the biological effects of pesticide exposure of the two cohorts, results found that a greater percentages of applicators had depressed levels of BChE than non-applicators (Fig 1). This is consistent with the findings of other studies [19–21]. While the relationship between neurological symptoms and BChE depression is obvious (Table 5), this is not the case regarding neurobehavioral performance, where only one test (Trail Making) showed a negative correlation with BChE levels in the 2005 cohort. This is similar to what was found among adolescent pesticide applicators in Egypt, where only Information, Digit Span and Trail Making were correlated with BChE activity [1]. These results also agree with findings from Roldan-Tapia and colleagues (2005), that BChE activity is not a valuable tool to explain neurological deficits among workers occupationally exposed to OP. Changes in BChE activity may be attributed to several factors e.g. inter- or intra-individual or seasonal variability or factors such as alcohol consumption [13].

The study was limited by the small sample size and only a single measurement of cholinesterase activity, which does not provide information about the inhibition of cholinesterase activity during the application season. Although other biomarkers of exposure are available to characterize exposure, e.g., TCPy, this information was only available for participants tested in 2009 [16]. Additional work examining the changes across the season is needed to understand the impact of exposure on neurological outcomes and to estimating the dose response relationship between pesticide exposure and neurobehavioral outcomes.

In conclusion, replicating the health findings associated with pesticide exposure among adolescent pesticide applicators tested in 2005 and 2009 in the Menoufia Governorate, Egypt demonstrates the neurological drawbacks and neurobehavioral deficits among adolescents occupationally exposed to OP pesticides. Neurological symptoms and signs were significantly higher among applicators than non-applicators, especially for the 2005 cohort. Fewer days and years worked in 2009 and also the small sample sizes may be the reasons that fewer neurological deficits were observed in 2009. These outcomes provide more evidence of the higher risk the adolescents may be exposed to when they work with dangerous chemicals such as pesticides. Due to the large number of children working in agriculture around the world, including those working on family farms [39], it is important to understand the impact of exposure on health outcome in order to change policy.

## Supporting information

S1 File Data file. Replication_Data_Block_Design101011.sav”. This is the raw data file.
(SAV)

S2 File Study questionnaire. EGAD Applicator Baseline Questionnaire.docx This is the questionnaire used for the current study.
(DOCX)

## Abbreviations

ANOVA: Analysis of Variance
BARS: Behavioral Assessment and Research System
BChE: Butyryl cholinesterase
M-H OR: Mantel-Haenszel common odds ratio (M-H OR)
OP: Organophosphorus
OR: Odds Ratio
WAIS-R: Wechsler Adult Intelligence Scale—Revised (WAIS-R)
