# Supplementary material for: Comparison of neurological health outcomes between two adolescent cohorts exposed to pesticides in Egypt
Source: PLoS One. 2017 Feb 23;12(2):e0172696. doi: 10.1371/journal.pone.0172696 (PMC5322908; doi:10.1371/journal.pone.0172696)
Supplement: S2 File — EGAD Applicator Baseline Questionnaire.docx This is the questionnaire used for the current study. (DOCX) [file pone.0172696.s002.docx]

Adolescent Project

Cotton Workers

**[ADD INSTRUCTIONS TO INTERVIEWER AND INSTRUCTIONS FROM INTERVIEWER TO PARTICIPANT]**

**A. OCCUPATIONAL HISTORY: APPLICATOR**

Thank you for your willingness to participate in our study. The purpose of the study is to understand better the health of working adolescents. Now, I am going to ask you several questions about your employment as an applicator for the Ministry of Agriculture.

A1. How many years, *including this year,* have you worked as a pesticide applicator for the Ministry of Agriculture?

[___ ___] years

A2. During pesticide application season, how many days do you usually work per week?

[___ ___] days

A3. During pesticide application season, how many hours do you usually work per day?

[___ ___] hours

A4. Do you usually mix the pesticide that will be applied to the fields?

[ ]……… No (0)

[ ]……… Yes (1)

A4a. If you usually mix pesticide, what do you use to mix it?

[ ]……… A stick (1)

[ ]……… Your hand (2)

[ ]……… Other (something not listed above) (specify: )

A5. Do you usually wear protective clothing or equipment when you personally handle or apply pesticides? (Mark all that apply)

| Personal Protective Equipment (PPE) | | Yes | No |
| --- | --- | --- | --- |
| A5a. | Shoes |  |  |
| A5b. | Head cover/cap |  |  |
| A5c. | Ear plugs or ear muffs |  |  |
| A5d. | Goggles over your eyes |  |  |
| A5e. | Glasses over your eyes for protection during spraying |  |  |
| A5f. | Mask over your mouth (not over your nose) |  |  |
| A5g. | Mask over your mouth AND nose |  |  |
| A5h. | Do you wear respirators? |  |  |
| A5i. | Name any other special work clothes you wear during pesticide applications |  |  |

A6. Did you apply pesticides to the cotton fields for the Ministry of Agriculture? (Check Yes or No for each year listed)

2006…...[ ]Yes (1)

[ ] No (0)

2007…...[ ]Yes (1)

[ ] No (0)

2008…...[ ]Yes (1)

[ ] No (0)

2009…...[ ]Yes (1)

[ ] No (0)

2010…...[ ]Yes (1)

[ ] No (0)

2011…...[ ]Yes (1)

[ ] No (0)

2012…...[ ]Yes (1)

[ ] No (0)

2013…...[ ]Yes (1)

[ ] No (0)

**B. Non-Ministry of Agriculture Pesticide Applications**

Now, I would like to ask you some questions about whether you have applied pesticides to agricultural fields as a private applicator. This does not include your work for the Ministry of Agriculture, but all other times you may have applied pesticides to agricultural fields. For example, you may have your own backpack sprayer and have been hired by other people to apply pesticides to crops such as vegetables.

B1. Have you ever applied pesticides to agricultural fields as a private applicator?

[ ]……… Yes(1)

[ ]……… No (0)

B1a. If you have applied pesticides as private applicator, how many times per week would you usually do this?

[___ ___] times per week

B1b. Do you know what pesticides you usually applied as a private applicator?

[ ]……. Yes(1) 🡪 if yes, list their names__________________________________

[ ]……. No (0)

B1c. What crops did you apply pesticides to as a private applicator (CHECK ALL THAT APPLY)?

[ ]……… Corn (1)

[ ] ………Cotton (2)

[ ] ………Vegetables (3)

[ ] ………Wheat (4)

[ ] ………Fruit (5)

[ ] ………Bersim (6)

[ ] ………Other (7) List_________________________________________

[ ] ………None (0)

B2. Do you plan to apply pesticides to crops as a private applicator this coming season?

[ ]……… Yes(1)

[ ]……… No (0)

**C. HOUSEHOLD, GARDEN, AND FAMILY FARM: USE OF PESTICIDES**

Now I am going to ask you some questions about the pesticides used at your home.[e.g. applying pesticides for mosquitoes or flies during the summer months], family garden and on your family farm, if you live on one.

C1. How many times in the past year have you sprayed for insects at your home?

[___ ___] Number

C2. Do other people in your house spray for insects at your home?

[ ]……… Yes (1)

[ ]…….. No (0)

C3. Does your family have a vegetable garden where vegetables are grown for your use?

[ ]……… Yes (1)

[ ] …….. No (0)

C3a. What crops are grown in your family garden?

[ ]……… Corn (1)

[ ] ………Cotton (2)

[ ] ………Vegetables (3)

[ ] ………Wheat (4)

[ ] ………Fruit (5)

[ ] ………Bersim (6)

[ ] ………None (0)

C4. Do you apply insecticides in your family garden?

[ ]……. Yes (1)

[ ]…….. No (0)

C5. Does your family have a farm/raise crops to sell?

[ ]……… Yes (1)

[ ] …….. No (0)

C5a. What crops are grown on your family farm?

[ ]……… Corn (1)

[ ] ………Cotton (2)

[ ] ………Vegetables (3)

[ ] ………Wheat (4)

[ ] ………Fruit (5)

[ ] ………Bersim (6)

[ ] ………None (0)

C6. Do you apply insecticides in your family farm?

[ ]……. Yes (1)

[ ]…….. No (0)

C7. Do you generally wear any special protective clothing or equipment when you personally handle or apply pesticides in your garden or family farm?

[ ]……… Yes (1)

[ ]…….. No (0)

If yes what type of special protective clothing or equipment do you wear when you personally mix or apply pesticides in your garden or family farm? **(Mark all that apply)**

| Personal Protective Equipment (PPE) | | Yes | No |
| --- | --- | --- | --- |
| B4a. | Shoes |  |  |
| B4b. | Head cover/cap |  |  |
| B4c. | Ear plugs or ear muffs |  |  |
| B4d. | Goggles over your eyes |  |  |
| B4e. | Glasses over your eyes for protection during spraying |  |  |
| B4f. | Mask over your mouth (not over your nose) |  |  |
| B4g. | Mask over your mouth AND nose |  |  |
| B4h. | Do you wear respirators? |  |  |
| B4i. | Name any other special work clothes you wear during pesticide applications |  |  |

**D. GENERAL QUESTIONS ABOUT YOUR HOME**

Now, I would like to ask you some questions about your home where you live and washing of your work clothes.

D1. How many people live in your home (not including yourself)?

[___ ___] Number of people

D2. Does anyone in your home smoke cigarettes?

[ ]……… Yes (1)

[ ]…….. No (0)

D2a. If yes, how many people in your home smoke cigarettes (not including yourself)?

[___ ___] Number of people

D2b. Does anyone in your home smoke cigarettes while you are home?

[ ]……… Yes (1)

[ ]…….. No (0)

D2c. If yes how many people in your home smoke cigarettes while you are home (not including yourself)?

[___ ___] Number of people

D3. Does anyone in your home smoke shisha?

[ ]……… Yes (1)

[ ]…….. No (0)

D3a. If yes, how many people in your home smoke shisha (not including yourself)?

[___ ___] Number of people

D3b. Does anyone in your home smoke shisha while you are home?

[ ]……… Yes (1)

[ ]…….. No (0)

D3c. If yes how many people in your home smoke shisha while you are home (not including yourself)?

[___ ___] Number of people

D4. How many people share your bedroom?

[ ]………Own room (0)

[ ]………1 person (1)

[ ]………2 persons (2)

[ ]………3 persons (3)

[ ]………4 persons (4)

D4a. If other people share your bedroom do any of them smoke in your bedroom?

[ ]………Yes (1)

[ ]………No (0)

C4b. If yes, how many people in your bedroom smoke cigarettes (not including yourself)?

[___ ___] Number of people

D5.Do you live in or within 25 meters of the edge of an agricultural field?

[ ]……… Yes (1)

[ ]…….. No (0)

D6. Who washes your work clothes?

[ ]………You (1)

[ }………Mother or sister (2)

[ ] ………Other family member (3)

D7. Where do you wash your work clothes?

[ ] ………Washing machine at home (1)

[ ] ………Sink at home (2)

[ ] ………Outside of home (3)

[ ] ………River (4)

D8. Do you keep your work clothes separate from your non-work clothes?

[ ]……… Yes (1)

[ ]…….. No (0)

D9. How many hours after work did you first wash your hands with soap after the application was completed yesterday?

│__││__│Hours

D10. How many hours after work did you first wash or bathe with soap after the application was completed yesterday?

│__││__│Hours

D11. How many hours after work did you change out of your work clothes after the application was completed yesterday?

│__││__│Hours

D12. Sometimes people wear the same clothes for several days before washing them, have your clothes been washed since you last applied?

│__│Yes (1)

│__│No (0)

D13. How often are your work clothes washed?

│__│Everyday (4)

│__│Every week (3)

│__│Every month (2)

│__│Once a year (1)

│__│Never (0)

**E . MEDICAL HISTORY**

Now, I am going to ask you about your medical history, including questions about your breathing and injuries.

E1. Have you ever been advised to change jobs or work assignments because of any health problem or injury?

[ ]………Yes (1)

[ ]………No (0)

E2. Have you had a history of any of the following? (Check all that apply)

[ ] ………nerve injury (1)

[ ] ………nerve disorder (2)

[ ] ………back injury (3)

E3. Have you ever gotten heat sickness?

[ ]……… Yes (1)

[ ]…….. No (0)

E4. Do you usually have a cough with colds?

[ ]………Yes (1)

[ ]………No (0)

E5. Do you usually have a cough apart from colds?

[ ]………Yes (1)

[ ]………No (0)

E5a.If yes, do you cough on most days (4 or more days per week) for as much as 3

months of the year?

[ ]………Yes (1)

[ ]………No (0)

E5b. For how many years have you had this cough?

[___ ___] Number of years

E6. Do you feel congested in the chest or bring up phlegm with colds?

[ ]………Yes (1)

[ ]………No (0)

E6a.If yes, do you bring up phlegm on most days (4 or more days per week) for as much as 3 months of the year?

[ ]………Yes (1)

[ ]………No (0)

E6b. For how many years have you brought up phlegm?

[___ ___] Number of years

E7. Do you get attacks of cough, chest congestion, or phlegm lasting for 1 week or more each year?

[ ]………Yes (1)

[ ]………No (0)

E7b.If yes, for how many years have you had these attacks?

[___ ___] Number of years

E7c. On average how many of these attacks do you have per year?

[___ ___] Number of attacks

E8. Does your chest ever sound wheezy or whistling when you have a cold?

[ ]………Yes (1)

[ ]………No (0)

E9. Does your chest ever sound wheezy or whistling apart from when you have a cold?

[ ]………Yes (1)

[ ]………No (0)

E9a. If yes, how often does your chest sound wheezy or whistling?

[ ]………Occasionally (1)

[ ]………Most days or nights (2)

E9b. If yes, for how many years has wheezing or whistling been present?

[___ ___] Number of years

E10. Do you ever have an attack of wheezing that caused you to be short of breath?

[ ]………Yes (1)

[ ]………No (0)

E10a. If yes, have you had 2 or more such episodes in the past year?

[ ]………Yes (1)

[ ]………No (0)

E10b. If yes to E11, for how many years have you had these episodes?

[___ ___] Number of years

E10c. If yes to E11, do you ever get these attacks of wheezing after you have been playing hard or exercising?

[ ]………Yes (1)

[ ]………No (0)

E11. During the past year have you had any chest illnesses that have kept you from your usual activities for at least 3 days?

[ ]………Yes (1)

[ ]………No (0)

E11a. If yes, how many illnesses like this have you had in the past year?

[___ ___] Number of illnesses

E11b. If yes, how many illnesses lasted for more than 7 days?

[___ ___] Number of illnesses

E12. Has a doctor ever said that you had an allergic reaction to food or medicine?

[ ]………Yes, food only (1)

[ ]………Yes, medicine only (2)

[ ]………Yes, food and medicine (3)

[ ]………No (0)

E13. Has a doctor ever said that you had an allergic reaction to pollen or dust?

[ ]………Yes (1)

[ ]………No (0)

E14. Has a doctor ever said that you had an allergic skin reaction to detergents or other chemicals?

[ ]………Yes (1)

[ ]………No (0)

E15. Have you ever received allergy shots?

[ ]………Yes (1)

[ ]………No (0)

E16. Has a doctor ever said you have asthma?

[ ]………Yes (1)

[ ]………No (0)

E16a. If yes to E17, at what age were you told you have asthma?

[___ ___] Age in years

E16b. If yes to E17, do you still have asthma?

[ ]………Yes (1)

[ ]………No (0)

E17b.2 If no to E17b, what age did your asthma stop?

[___ ___] Age in years

E16c. Do you currently take medicine or treatment for asthma?

[ ]………Yes (1)

[ ]………No (0)

E17. If yes to E17c, please list the medication you take to control asthma:

1)_____________________________________

2)_____________________________________

3)_____________________________________

4)_____________________________________

**F. SYMPTOMS**

Now, I am going to ask you about how you physically felt in the past three months.

F1. How often during the past 3 months have you experienced the following symptoms?

| About how often during the past 3 months have you experience the following symptoms: | More than once | Once | Never |  |
| --- | --- | --- | --- | --- |
| 1. Dizziness | [ ] | [ ] | [ ] | |
| 2. Feeling tense, anxious, or nervous | [ ] | [ ] | [ ] | |
| 3. Nausea/ Vomiting | [ ] | [ ] | [ ] | |
| 4. Feeling tired, sleepy, or low energy most of the day | [ ] | [ ] | [ ] | |
| 5. Sweating a lot more than usual | [ ] | [ ] | [ ] | |
| 6. Difficulty seeing at night | [ ] | [ ] | [ ] | |
| 7. Being absentminded, forgetful or confused | [ ] | [ ] | [ ] | |
| 8. Headache | [ ] | [ ] | [ ] | |
| 9. Loss of appetite | [ ] | [ ] | [ ] | |
| 10. Fast heart rate | [ ] | [ ] | [ ] | |
| 11. Difficulty with balance | [ ] | [ ] | [ ] | |
| 12. Blurred vision or double vision | [ ] | [ ] | [ ] | |
| 13. Difficulty concentrating | [ ] | [ ] | [ ] | |
| 14. Numbness or pins-and-needles in your hands and feet | [ ] | [ ] | [ ] | |
| 15. Momentary loss of consciousness | [ ] | [ ] | [ ] | |
| 16. Feeling excessively irritable or angry | [ ] | [ ] | [ ] | |
| 17. Shaking or trembling of your hands | [ ] | [ ] | [ ] | |
| 18. Difficulty falling asleep or staying asleep | [ ] | [ ] | [ ] | |
| 19. Difficulty speaking | [ ] | [ ] | [ ] | |
| 20. Weakness in your arms or legs | [ ] | [ ] | [ ] | |
| 21. Changes in your sense of smell or taste | [ ] | [ ] | [ ] | |
| 22. Feeling depressed, indifferent or withdrawn | [ ] | [ ] | [ ] | |
| 23. Twitches, jerks or involuntary movements of your arms or legs | [ ] | [ ] | [ ] | |
| 24. Excessive salivation | [ ] | [ ] | [ ] | |
| 25. Tinnitus or ringing in your ears | [ ] | [ ] | [ ] |  |

F2. How many episodes of wheezing or whistling in your chest have you had in the past 12 months?

[___] Times

F3. During the past 12 months, how many times have you gone to the emergency room or doctor’s office for an episode of wheezing or whistling?

[___] Time

**G. LIFESTYLE ACTIVITIES**

Now, I am going to ask you some questions about your lifestyle activities.

G1. Have you ever smoked cigarettes?

[ ] ………Yes (1)

[ ] ………No (0)

G1a. If yes, when did you start?

[ ] less than 6 months ago (1)

[ ] 6-12 months ago (2)

[ ] more than 1 year ago (3)

[ ] more than 2 years ago(4)

[ ] more than 3 years ago (5)

G1b. Do you smoke cigarettes now?

[ ]……… Yes (1)

[ ]…….. No (0)

G1c. What is the total number of months you smoked cigarettes? (Remember to leave out months that you did not smoke)

[___ ___]Months

G1c. On a usual day how many cigarettes do you smoke? (1 pack=20 cigarettes)

[ ]……… 10 cigarettes or less (1)

[ ]……… 11-20 cigarettes (2)

[ ]……… 21-40 cigarettes (3)

[ ]………More than 40 cigarettes (3)

G2. Have you ever smoke SHISHA?

[ ]……… Yes (1)

[ ]…….. No (0)

G2a. If yes, how many times do you smoke SHISHA per day?

[___ ___] Times per day

**H. GENERAL INFORMATION**

Thank you very much for participating in the EGAD study. Before you move on to the next part of your visit, I have a couple of general questions for you and your family.

H1. What is today’s date?

[___ ___] Day

[___ ___] Month

[____ ____ ____ ___] Year

H2. How many years of school have you completed?

[___ ___] Years

H3. How often do you use a computer?

[ ]………Everyday (6)

[ ]………2 or 3 times a week (5)

[ ]………Once a week (4)

[ ]………Once every couple of weeks (3)

[ ]………Once a month (2)

[ ]………Once every couple of months (1)

[ ]……… Never (0)

H4. What is your father’s highest level of education attained?

[ ]………Primary school (1)

[ ]………Preparatory school (2)

[ ]………Secondary school (general & technical of 3 to 5 years) (3)

[ ]………Intermediate (2 years) institutes (4)

[ ]………University graduate (5)

[ ]………Postgraduate degree (6)

H5. What is your mother’s highest level of education attained?

[ ]………Primary school (1)

[ ]………Preparatory school (2)

[ ]………Secondary school (general & technical of 3 to 5 years) (3)

[ ]………Intermediate (2 years) institutes (4)

[ ]………University graduate (5)

[ ]………Postgraduate degree (6)

H6. Is your address_________________________________________________(family domain)

H7. How would you describe your household income (from all sources)

[ ]………Inadequate (1)

[ ]………Just enough to meet routine expenses (2)

[ ]………Enough to meet routine expenses and emergencies (3)

[ ]………Enough to save money (4)

H8. Does your family receive governmental support?

[ ]……… Yes (1)

[ ]…….. No (0)

H9. Does your family pay income tax?

[ ]……… Yes (1)

[ ]…….. No (0)

H10. How would you categorize your father’s occupation

[ ]………Not working (1)

[ ]………Unskilled manual worker (2)

[ ]………Skilled manual worker (3)

[ ]………Farmer (4)

[ ]………Trade (5)

[ ]………Semi-professional (example, store clerk) (6)

[ ]………Professional (7)

H11. How would you categorize your mother’s occupation

[ ]………House wife (1)

[ ]………Unskilled manual worker (2)

[ ]………Skilled manual worker (3)

[ ]………Farmer (4)

[ ]………Trade (5)

[ ]………Semi-professional (example, store clerk) (6)

[ ]………Professional (7)

H12. Does your family own any of the following? (check all that apply)

[ ]………Refrigerator (1)

[ ]………Radio (2)

[ ]………Television (3)

[ ]………Washing machine (4)

[ ]………Telephone/mobile phone (5)

[ ]………Car (6)

[ ]………Agricultural land (7)

[ ]………Non-agricultural land for housing (8)

[ ]………Shop or animal shed (9)

[ ]………Other house (besides the house in which your family lives in) (10)

[ ]………Animals (example, chickens) (11)

[ ]………Computer with internet (12)

H13. Does your house have any of the following services?

[ ]………Pure water supply (1)

[ ]………Electricity (2)

[ ]………Natural gas(3)

[ ]………Sewage system (4)

[ ]………Municipal collection of solid waste (5)

[ ]………Flush latrine (6)

[ ]………Air conditioning (7)

H14. What type of house do you live in?

[ ]………Owned with 4 or more rooms (1)

[ ]………Owned with less than 4 rooms (2)

[ ]………Rented with 4 or more rooms (3)

[ ]………Rented with less than 4 rooms (4)

[ ]………No place to reside (5)

H15. How many people, including yourself, live in your home?

[___ ___] number of people

H16. What is your usual source of health care?

[ ]………Private health care facility (1)

[ ]………Health Insurance (2)

[ ]………Free governmental health care service (3)

[ ]………Traditional healer (4)

[ ]………Self-care (5)
